# Prehydrated collagenated cortico‐cancellous heterologous bone gel and papillae tunneling for isolated intrabony defects: 12‐month noninferiority trial

**DOI:** 10.1002/cre2.853

**Published:** 2024-02-06

**Authors:** Tom Kobe, Katja Povšič, Rok Gašperšič

**Affiliations:** ^1^ Department of Oral Medicine and Periodontology University Medical Centre Ljubljana Slovenia; ^2^ Department of Oral Medicine and Periodontology, Faculty of Medicine University of Ljubljana Ljubljana Slovenia

**Keywords:** dual‐phase xenogenic bone graft, intrabony defect, papillae preservation, periodontitis

## Abstract

**Objectives:**

This study assessed the effectiveness of prehydrated collagenated xenogenic bone gel and a collagenated cortico‐cancellous heterologous bone mixture in conjunction with papillae tunneling techniques (PTT) for treating isolated periodontal intraosseous defects.

**Materials and Methods:**

Twenty patients with periodontitis stage III/IV and at least one deep isolated interdental 2/3‐wall intraosseous defect were included in the study. Surgical incisions were made vertically at the adjacent tooth or horizontally at the mucogingival junction. A full‐thickness flap was then carefully lifted under the papillae using special tunneling instruments. The root surfaces were completely cleaned, and the defects were randomly filled with either prehydrated collagenated bone gel (test group; *n* = 10) or collagenated cortico‐cancellous heterologous bone mixture (control group; *n* = 10). Wounds were closed with microsurgical sutures. We predicted that the lower 95% confidence interval for the difference between the two procedures would exceed a prespecified noninferiority threshold.

**Results:**

All wounds closed sufficiently to prevent biomaterial exposure. The test and control groups showed similar mean pocket depth reduction (3.5 ± 1.0 vs. 3.9 ± 1.7 mm; *p* = 0.52), similar gingival recession (−0.10 ± 0.99 vs. 0.2 ± 0.8 mm; *p* = 0.46), and similar clinical attachment gain (3.6 ± 1.51 vs. 3.7 ± 1.8 mm; *p* = 0.89) at the 12‐month follow‐up. All results were below the noninferiority margin of the sample.

**Conclusions:**

At 12 months, prehydrated collagenous bone gel performed similarly to collagenous heterologous bone granules in the treatment of intraosseous lesions with PTT. In addition, both biomaterials preserved soft tissue with minimal further recession at 1 year.

**Clinical Relevance:**

When combined with PTT, collagenous xenogeneic bone granules and prehydrated collagenous bone gel achieve comparable clinical outcomes in intrabony defects. The study was registered under the NCT 04782921 on ClinicalTrails.

## 1 INTRODUCTION

Periodontitis is an inflammatory disease that leads to progressive loss of periodontal tissue characterized by radiographic bone loss or clinical attachment loss (CAL) as measured by probing (Papapanou et al., 2018). Adequate treatment involves a stepwise approach depending on the stage of disease, primarily aimed at reducing the amount of supra‐ and subgingival plaque, calculus deposits, and periodontal bacterial load (Sanz et al., 2020). It initially involves behavioral changes in oral hygiene, control of modifiable risk factors, and nonsurgical debridement procedures, followed by extractions and/or surgical interventions at nonresponsive sites (Herrera et al., 2022). Resective surgical approaches offer limited potential for clinical and radiographic restoration of lost periodontal tissue. Therefore, regenerative approaches combined with minimally invasive surgical techniques are often used instead, especially in the treatment of deep intrabony defects (Al Machot et al., 2014), which pose a high risk for periodontitis progression and tooth loss (Nieri et al., 2002). Minimally invasive surgical techniques based on conservative flap designs minimize the extent of flap reflection and wounding and improve coagulation stability, primary closure, and space preservation. They also offer advantages in patient‐oriented outcomes: reduced postoperative morbidity, shorter recovery times, and preservation of preexisting gingival esthetics (Kao et al., 2015). However, when traditional papilla‐preserving techniques, xenogeneic grafts, and resorbable membranes are used, primary wound closure (WC) can be maintained in the early healing phase in only about half of the cases, often resulting in biomaterial exposure (Cortellini et al., 2001).

Two surgical methods, the Entire Papilla Preservation Technique (EPP [Aslan et al., 2017]) and the Nonincised Papillae Surgical Approach (NIPSA [Moreno Rodriguez & Caffesse, 2018]), have recently been developed to avoid incising of the interdental papillae when treating deep intrabony defects. To gain direct access to intrabony defects with NIPSA, a horizontal incision is made through the attached gingiva above the marginal bone level and a small, full‐thickness flap is elevated in a coronal direction. The papillary tissue is left in its original position as a dome‐like protection. When using the EPP technique, a short vertical incision is often made at an adjacent tooth or along the contralateral line angle of the same tooth, and then the papilla is tunneled from the lateral side. After the removal of granulation tissue, the tooth root surface facing the intrabony defect must be thoroughly debrided. The defect is then filled with a slow resorbing particulate bone graft material in compliance with the original procedural instructions. In the final step, the incision line is sutured. While NIPSA seems to be more suitable for the anterior and maxillary region, EPP should be avoided in the anterior region because of the possible scarring (Pei, 2021). A common aspect of EPP and NIPSA is a modified nonsurgical approach (level 2) that does not involve aggressive scaling and root planing at sites with deep intrabony defects.

To prevent soft tissue collapse and improve papilla support, slow resorbing dual phase collagenated particulate bone graft substitutes were used in the original descriptions of EPP (Aslan et al., 2017, 2021) and xenogenic bone without organic particles in the original descriprion of NIPSA (Moreno Rodriguez & Caffesse, 2018). However, a slow resorbing bone graft substitute may interfere with wound healing and periodontal regeneration. Recently, both EPP (Aslan et al., 2020) and NIPSA (Moreno Rodríguez & Ortiz Ruiz, 2022) were investigated in randomized controlled clinical trials comparing xenogeneic bone grafts with surgery alone (without bone graft). These trials did not clearly demonstrate the benefits of bone graft substitutes in combination with NIPSA (Moreno Rodríguez & Ortiz Ruiz, 2022) or EPP (Aslan et al., 2020), as the same clinical outcomes in terms of periodontal regeneration were obtained regardless of the application of bone graft substitutes to the defect sites. In addition, Moreno Rodriguez and Ortiz Ruiz (2022) reported that deeper residual pockets and lower levels of new attachment formation were generally observed after the use of slow resorbing bone substitutes; however, the bone substitutes improved interproximal soft tissue volume.

The use of dual phase xenogenic bone substitutes containing collagen, which acts as a signaling molecule, simultaneously provides a scaffold for bone regeneration and stimulates natural healing processes (Falacho et al., 2021). However, the ability of dual phase xenogeneic bone gels to maintain papillae is currently unknown and may be questionable due to the smaller size and lower proportion of bone particles, which lack the mechanical properties to support soft tissue. To promote optimal healing and prevent papilla collapse, we aimed to evaluate the clinical performance of a prehydrated collagen‐containing xenogenic bone gel that contains a lower proportion of smaller bone particles than a standard bone graft. Given the widely recognized efficacy of slow resorbable particulate bone grafts used in conjunction with regenerative papillary tunneling procedures, we developed a protocol for a noninferiority study designed to demonstrate comparable success in clinical parameter improvement following the utilization of xenogenic bone gel. Our hypothesis posited that the lower 95% confidence interval (CI) for the differences between the baseline and 1‐year results would surpass a pre‐established threshold for noninferiority.

## 2 MATERIALS AND METHODS

### 2.1 Study design and population

The study was designed as a randomized, controlled, single‐center clinical trial. Ethical approval was obtained from the National Medical Ethics Committee (protocol no. 0120‐653/2017/3), and the study was registered on ClinicalTrials.gov (NCT04782921). Before participation, all patients consented by signing appropriate forms. The study was conducted in accordance with the tenets of the Declaration of Helsinki.

Twenty individuals were meticulously chosen from a pool of 86 consecutively assessed patients seeking periodontal treatment at the Department of Oral Medicine and Periodontology, University Medical Center Ljubljana, Ljubljana, Slovenia. This selection process was conducted between January 2020 and 2021. The inclusion criteria were: patients diagnosed with stage Ill/IV periodontitis (Tonetti et al., 2018); at least one isolated deep 3‐wall intrabony defect with a partial 2‐wall component on the periodontally affected tooth (Papapanou & Tonetti, 2000); predominant involvement of the interproximal region of the affected tooth with probing depth (PD) ≥ 5 mm and clinical attachment level (CAL) ≥ 6 mm; full‐mouth plaque score (FMPS) [17]; and full‐mouth bleeding score (FMBS) below 20% [18].

The exclusion criteria included heavy smokers (more than 10 cigarettes per day), patients with known systemic diseases, and pregnant or lactating women. In addition, inadequately endodontically treated teeth were also excluded from the study.

### 2.2 Initial treatment

All subjects underwent an initial phase of nonsurgical periodontal therapy, which included education and instruction on proper oral hygiene, removal of supra‐ and subgingival deposits with piezoelectric ultrasonic instruments (PiezoLED ultrasonic scaler with Piezo Scaler tip 203 [KaVo Dental]), followed by scaling and root planing of sites with a PD of ≥5 mm, performed under local anesthesia (Ultracain©) with Gracey curettes (Hu‐Friedy).

### 2.3 Surgical procedure

**Figure 1:**
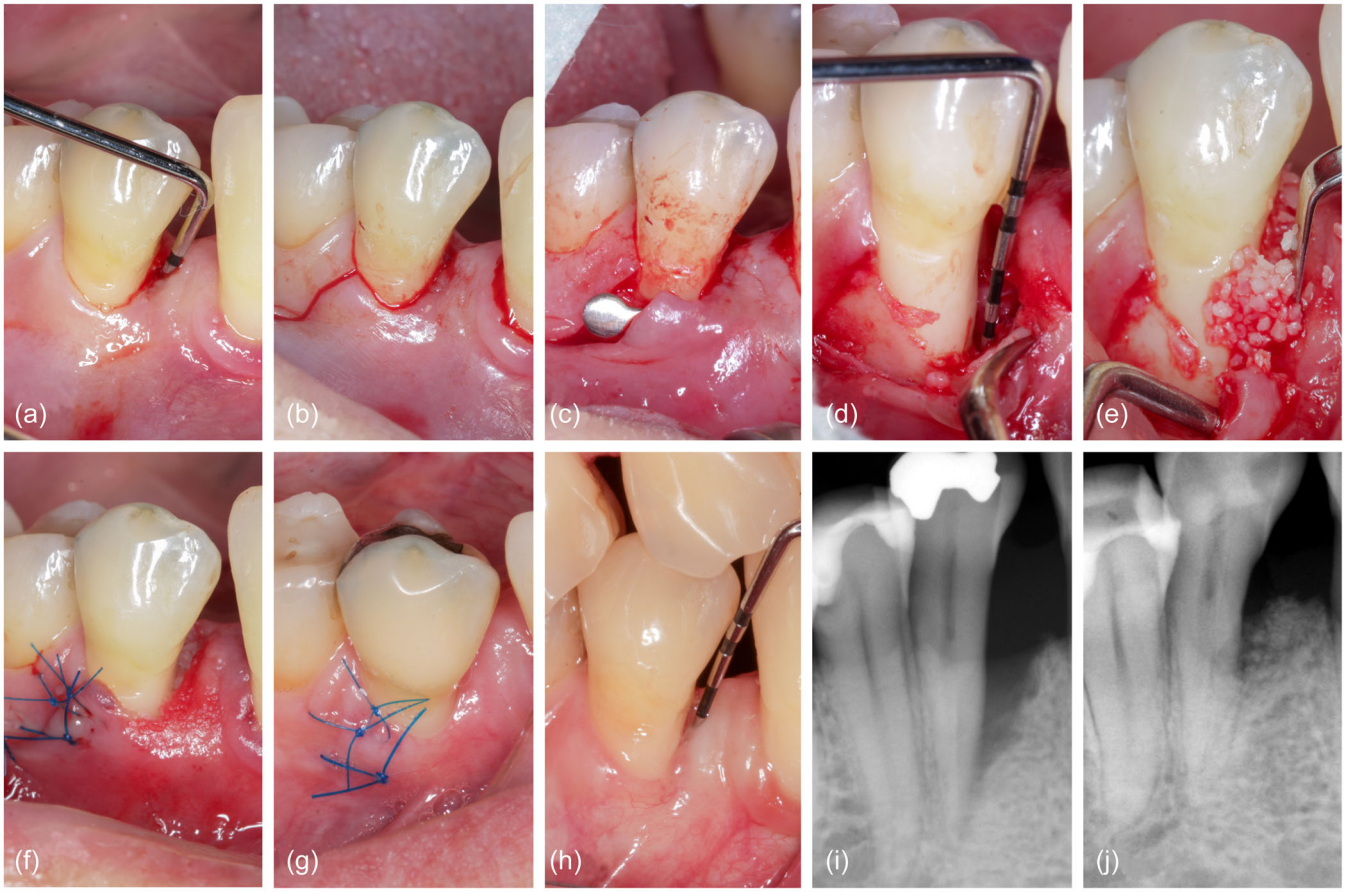
Representative case from the control group, treated with EPP and xenogenic bone mix. (a) Baseline measurement, (b) single vertical incision, (c) reflection of a small mucoperiosteal flap, (d) granulation tissue removal, (e) application of the standard slow‐resorbing bone substitute, (f) suturing, (g) healing after 1 week, (h) measurement after 1 year, (i) baseline radiograph, (j) 1‐year radiograph. EPP, entire papilla preservation technique.

**Figure 2:**
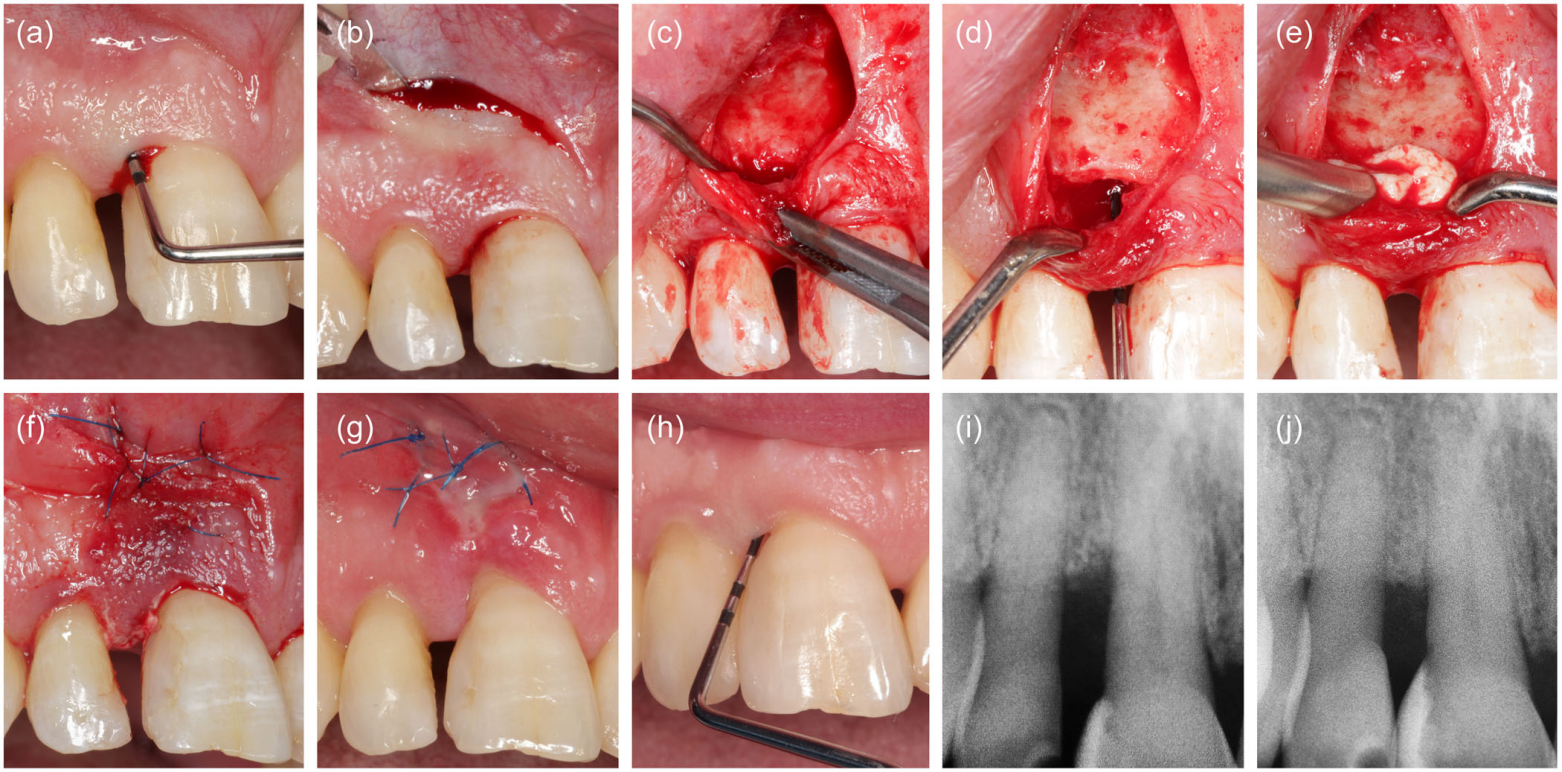
Representative case from the test group, treated with NIPSA and prehydrated collagenated bone mix. (a) Baseline measurement, (b) single horizontal incision, (c) inter‐dental tunnel preparation, (d) granulation tissue removal, (e) application of material, (f) primary closure of surgical area, (g) healing after 1 week, (h) measurement after 1 year, (i) baseline radiograph, (j) 1‐year radiograph. NIPSA, nonincised papillae surgical approach.

**Figure 3:**
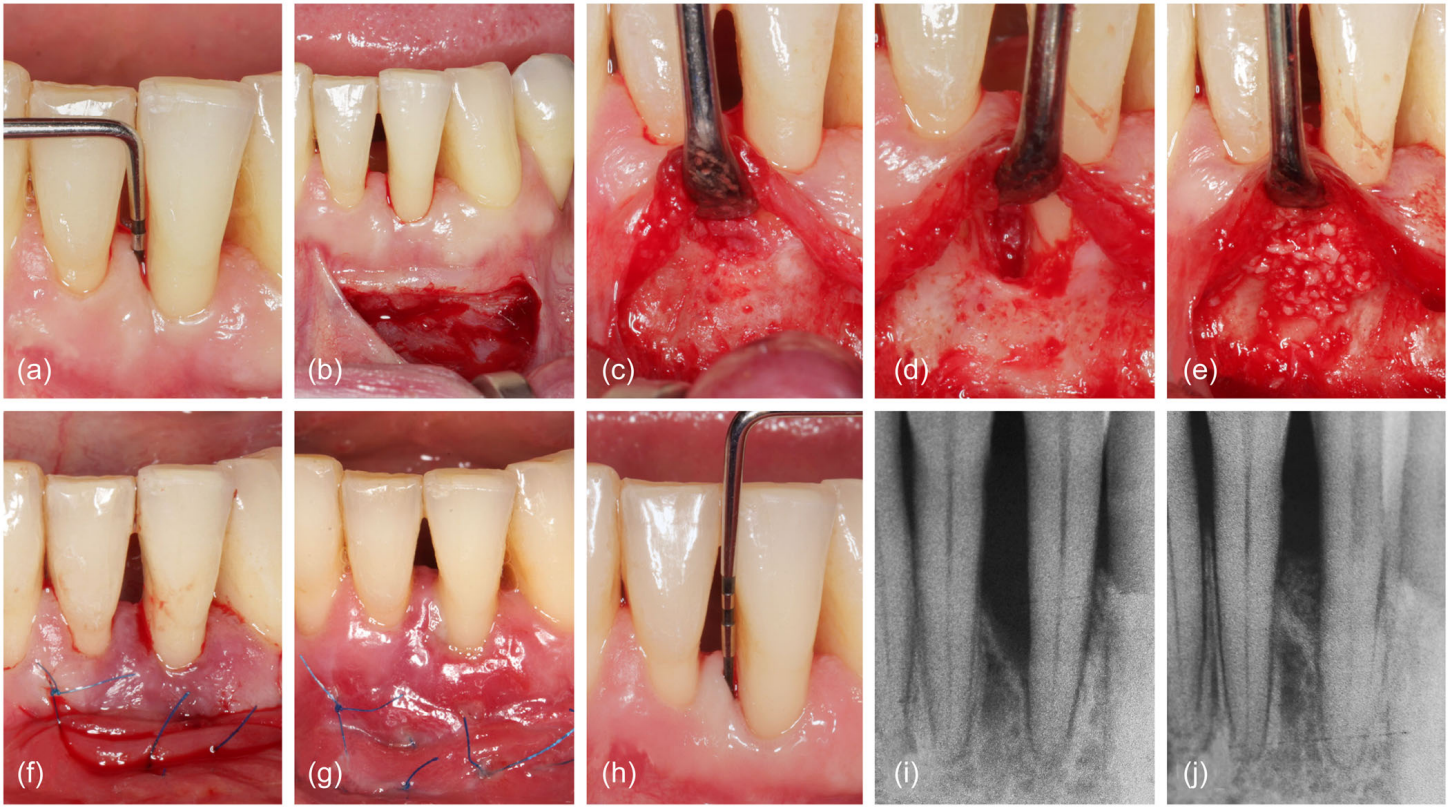
Representative case from the control group, treated with NIPSA and xenogenic bone mix. (a) Baseline measurement, (b) single horizontal incision, (c) reflection of a small mucoperiosteal flap, (d) granulation tissue removal, (e) application of the standard slow‐resorbing bone substitute, (f) suturing, (g) healing after 1 week, (h) measurement after 1 year, (i) baseline radiograph, (j) 1‐year radiograph. NIPSA, nonincised papillae surgical approach.

Three months after the initial nonsurgical periodontal therapy, patients were invited for a follow‐up appointment. Interproximal sites with PD ≥ 5 mm and CAL ≥ 6 mm and associated intrabony defects were selected for the study (Figures 1, 2 and 3a) (Sanz et al., 2020).

Twenty surgical treatments were performed by a single resident of periodontology (T. K.). Surgical incisions were chosen to protect the integrity of the interdental papillae and to take anatomical factors into account. In this regard, defects associated with incisors and canines were accessed using NIPSA (*n* = 11) (Aslan et al., 2017), whereas EPP was performed at defects associated with premolars or molars (*n* = 9) (Moreno Rodriguez & Caffesse, 2018).

After administration of local anesthetic (Ultracain©), an intracrevicular incision was made around the teeth affected by the defect. Then, either a short vertical incision was made in the buccal gingiva just beyond the mucogingival line as part of EPP (Figure 1b) (Aslan et al., 2017) or a single horizontal apical incision was made in the mucosa apically to the edge of the bony ridge bordering the defect as part of NIPSA (Figures 2b and 3b) (Moreno Rodriguez & Caffesse, 2018). After elevation of a buccal, mucoperiosteal, full‐thickness flap, a tunneling instrument was used to undermine a tunnel preparation at the defect‐associated papilla (Figures 1, 2 and 3c). The granulation tissue was excised using microsurgical scissors and a tiny curette (Micro Mini Five Gracey Curette; Hu Friedy) (Figures 1, 2 and 3d). The exposed root surface was cleaned with an ultrasonic scaler (NSK Varios 980; NSK Dental), and a small curette was used to remove subgingival calculus or plaque. The surgical site was carefully cleaned with sterile saline, and the treatment was continued according to the random assignment protocol.

In the test group (*n* = 10), a prehydrated collagen‐containing corticocellular heterologous bone gel (OsteoBiol® Gel 40; granulometry up to 300 μm) was applied to the exposed root surface using a syringe (Figure 2e). In the control group (*n* = 10), the intraosseous defect was filled with the patient's blood‐soaked collagenous corticocellular xenogeneic bone graft (Figures 1e and 3e) (OsteoBiol® Gen‐Os; granulometry from 250 to 1000 μm). During application, contamination of the root surface with saliva was prevented by relative isolation. Microsurgical suture techniques with 6–0 or 7–0 monofilament sutures (Prolene; Ethicon) were used for WC (Figures 1, 2 and 3f), and gentle pressure was applied to the surgical site with saline‐moistened gauze for 1 min after completion of surgery for mucoperiosteal flap adaptation.

Patients were instructed to refrain from oral hygiene in the surgical area for 3 weeks and instead rinse twice daily with 0.12% chlorhexidine digluconate (Curasept; Curaprox). One week after surgery, patients were asked to report side effects and problems (Figures 1, 2, and 3g). Sutures were removed 2 weeks after the procedure.

As part of the maintenance protocol, T. K. performed a professional dental cleaning on each patient every 3 months for the next 12 months.

### 2.4 Clinical parameters

Three months after the initial periodontal treatment and 1 year after the surgical intervention, clinical periodontal parameters were recorded (Figures 1, 2 and 3h). All clinical measurements at baseline and 1 year were performed with a manual Williams probe (POW6; Hu‐Friedy) by the same experienced investigator who was blinded to the assignment to the research group (R. G.). A calibration exercise with measurements repeated after 1 week yielded intraclass correlation coefficients for PD and CAL above 0.9 and *ϰ* values for PlI, and bleeding on probing (BOP) above 0.95, indicating excellent reproducibility.

FMPS and FMBS were recorded at baseline. PD and gingival recession (REC) at the deepest part of the experimental area were rounded to the nearest millimeter (POW6; Hu‐Friedy). CAL was calculated by adding the values of PD and REC. In addition, BOP was scored using a binary scale, with scoring performed 15 s after probing. During the procedure, the intraosseous component (INTRA), that is, the distance between the bone crest and the deepest part of the bony defect, and the intraosseous component with three walls (3W) were measured.

Intraoral photographs were used to determine the location of the papillae (TP) for comparison of the initial and final conditions. The manual Williams probe was used to calibrate the scale, and the position TP was measured relative to the incisal edge of the adjacent tooth (ImageJ; NIH). Each measurement was taken three times and then averaged to obtain a single value.

Primary surgical site closure was assessed after 2 weeks and characterized as either complete WC = 2, incomplete closure with a fibrin cloth (WC = 1), or biomaterial exposure (WC = 0) (Table 3). Radiographic images at baseline (Figures 1, 2 and 3i) and 1 year after therapy (Figures 1, 2 and 3j) were used for radiological analysis. Distances were determined by projecting three reference points onto a straight line constructed along the long axis of the tooth: the enamel–cement interface, the most apical point of the defect (before and after treatment), and the apex of the tooth. The change in radiographic bone height as a function of root length was then calculated. The radiographic periodontal defect angle was also measured (Cortellini & Tonetti, 2011).

### 2.5 Sample size and randomization

Sample size was determined using CAL gain as the primary outcome. A difference of 1.5 mm in CAL gain was assumed to be clinically significant (Aslan et al., 2020); expected variation was set at 1.1 mm (Cortellini & Tonetti, 2011). With *α* = 0.05 and power of 80%, the calculated sample size was 20 surgical sites (10 per group).

The selected single intraosseous defect of each patient was randomly assigned to one of the two biomaterials. Numbered and opaque sealed envelopes were used to conceal the assignment. The random sequence was generated using a computer‐generated randomization table (Microsoft Excel; Microsoft Corporation). The opaque sealed envelopes were opened immediately after defect debridement, and the operator was informed of the biomaterial allocation (T. K.). The investigator (R. G.) remained blind throughout the study.

### 2.6 Noninferiority margin

The noninferiority of the test intervention was determined using a minimum standardized difference detectable in this study (d) as the margin, with the equivalence interval between −d and d. Considering the sizes of both groups, it was calculated that an expected effect size of *d* = 1.32 could be detected with 80% power and 5% significance level (Cohen, 2013). In accordance with the fixed margin approach, a size equal to the expected effect size of the active comparator was chosen as the margin (*M* = 1.32) to ensure the efficacy of the new treatment. For each outcome, noninferiority was demonstrated if the CI was completely above the lower margin of the equivalence interval, −d.

### 2.7 Statistical analysis

Demographic variables were described as means with standard deviations (SD) or counts and proportions (%), as appropriate. Outcome variables were changes in CAL gain (diffCAL), PD (diffPD), REC (diffREC), and TP (diffTP). Data were reported as mean values (SD) for each group. Cross‐group comparisons were performed as univariate analyses. *p* Values were determined using the two‐tailed independent *t*‐test with Welch correction. The standardized mean difference by Cohen's *d* and its CI were calculated for all outcomes as effect sizes for the efficacy of the main exposure compared with the gold standard. Analysis was performed using R statistical software, version 4.2.1, at *α* = 0.05.

### 2.8 Ethics statement

Approval No. 0120‐653/2017/3 was granted by the National Medical Ethics Committee of the Republic of Slovenia to conduct the research. All procedures involving human participants followed the ethical standards of the 1964 Helsinki Declaration and the Code of Medical Ethics of the Medical Association of Slovenia. Patients signed an informed consent form before treatment.

## 3 RESULTS

**Figure 4:**
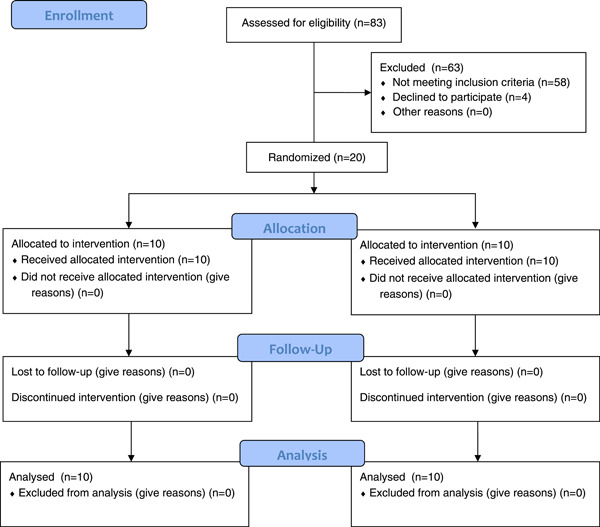
Consort flow diagram.

Twenty of the 83 examined patients were selected as participants for this study. Ten participants were randomly assigned to each group, and all were regularly recalled until the 1‐year follow‐up. Data from all 20 participants were included in the final analysis (Figure 4).

### 3.1 Study population and clinical characteristics

A total of 20 subjects participated in the study, of whom 12 (60%) were women. The mean age (SD) of the study participants was 53 (9) years. The test and control groups did not differ in any of the baseline clinical parameters (Table 1).

**Table 1:** Patient characteristics and clinical parameters measured at baseline.

	Gel‐40 (*n* = 10)	Gen‐Os (*n* = 10)	*p* Value
Study population			
Sex (male/female)	1/9	5/5	0.140^a^
Age (years) [mean ± SD]	54.5 ± 7.0	52.3 ± 10.2	0.853^b^
Smoking (nonsmokers/smokers)	8/2	8/2	1.000^a^
Dental arch (maxillary/mandibular)	4/6	3/7	0.500^a^
Tooth type (incisors/canines/premolars/molars)	3/4/2/1	3/1/2/4	0.372^a^
KT width (mm) [mean ± SD]	4.3 ± 1.2	3.2 ± 1.5	0.123^b^
Periodontal defect characteristics			
CEJ‐defect bottom (mm) [mean ± SD]	9.5 ± 1.3	10.1 ± 2.5	0.481^b^
Intraosseous component (mm) [mean ± SD]	4.5 ± 1.4	4.6 ± 1.3	0.870^c^
3‐wall component (mm) [mean ± SD]	2.0 ± 0.5	1.8 ± 0.8	0.529^b^
Main defect configuration (−1/−2/−3 wall)	1/4/5	1/7/2	0.475^a^
X‐ray angle (deg.) [mean ± SD]	31.9 ± 9.1	33.7 ± 11.8	0.853^b^
PD (mm) [mean ± SD]	7.5 ± 1.0	8.0 ± 1.2	0.390^b^
REC (mm) [mean ± SD]	2.0 ± 0.6	2.1 ± 1.6	0.600^b^
CAL (mm) [mean ± SD]	9.5 ± 1.2	10.1 ± 2.4	0.480^b^
Surgery type (NIPSA/EPPT)	7/3	4/6	0.370^a^

Abbreviations: CAL, clinical attachment loss; CEJ, cemento enamel junction; KT, keratinized tissue; NIPSA, nonincised papillae surgical approach; PD, probing depth; REC, gingival recession; SD, standard deviation.

^a^ Fisher's exact test.

^b^ Independent‐samples Mann–Whitney *U* test.

^c^ Independent‐samples *t*‐test.

### 3.2 Clinical outcome change distribution

**Figure 5:**
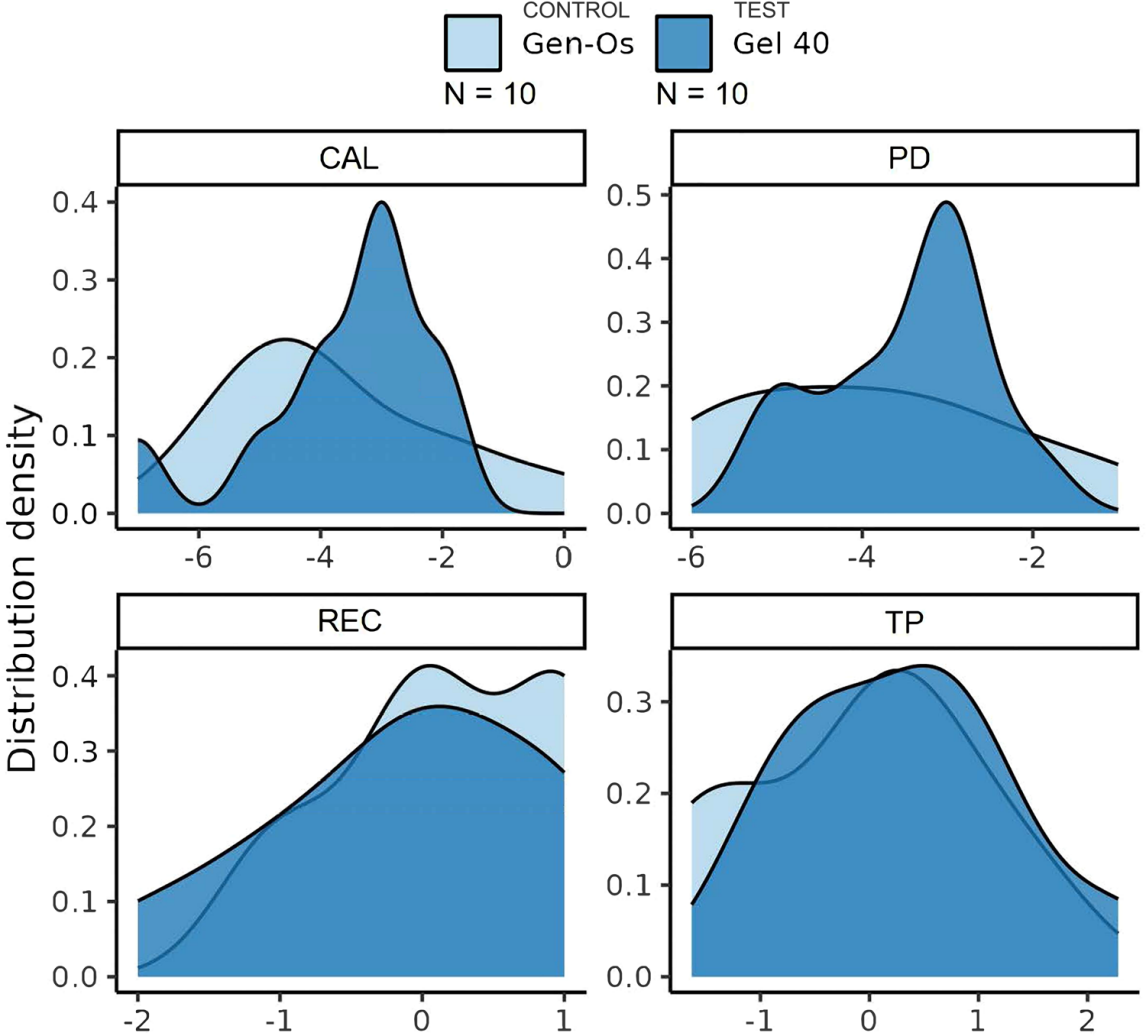
Density distribution of changes in study outcomes. CAL, clinical attachment loss; PD, probing depth; REC, gingival recession; TP, location of papilla.

The distribution density of the change in outcome levels is shown in Figure 5. The center of change of CAL was slightly higher in the test group than in the control group, but the variability in the control group was much greater, as can be seen from the width of the distribution, and with a strong tail toward higher values. The distribution of diffCAL in the test group appears to be bimodal, although its shape appears to be more clearly defined, with less variability around the center. This could be due to a larger number of participants being in the lower range of diffREC compared with the control group. The diffPD distribution appears to be slightly bimodal in the test group; the average change was higher than in the control group, but this could be due to the small sample size. The diffREC distribution has similar average values in both groups, but as mentioned earlier, the test group has a stronger tail in the lower part of the data. The distributions of diffTP in both groups appear to be similar in center, width, and shape.

### 3.3 Inferential analysis

After 1 week, complete WC was observed in almost all cases; only one case in the control group and three cases in the test group healed with fibrin cloth covering (N. S.). Exposure of the biomaterial never occurred (Table 2). The difference in changes in outcomes after 12 months of healing for both interventions is shown in Table 3. The mean (SD) diffCAL was −3.6 (1.5) mm in the test group and −3.7 (1.8) mm in the control group (*p* = 0.895). This corresponds to a standardized mean difference of *d* = −0.06 (95% CI: −0.94 to 0.82). The standardized effect of treatment on change was *d* = 0.33 (95% CI: −0.55 to 1.20) for diffREC, *d* = −0.29 (95% CI: −1.20 to 0.59) for diffPD, and *d* = −0.38 (95% CI: −1.30 to 0.51) for diffTP. Noninferiority was defined for this analysis by the lower range of the 95% CI being greater than *d* = 1.32. All results met this criterion; the noninferiority of the tested material was within the limits of the sample. In addition, bone fill of the test group (51%) was not significantly lower than that of the control group (53%) (*p* = 0.580) (Table 2).

**Table 2:** Wound closure and change in radiographic bone height.

	Gel‐40 (*n* = 10)	Gen‐Os (*n* = 10)	*p* Value
Would closure (0/1/2)	0/3/7	0/1/9	0.580^a^
Change in bone height (%)	51.4 ± 28.3	53.4 ± 28.9	0.87^b^

^a^ Fischer's exact test.

^b^ Independent samples *t*‐test.

**Table 3:** Effect of the OsteoBiol^©^ Gel 40 intervention in the average change of CAL, REC, PD, and TP in mm.

	Gel‐40 (*n* = 10)	Gen‐Os (*n* = 10)	*d*	95% CI	*p* Value
diffPD (mm) [mean ± SD]	−3.50 ± 0.97	−3.90 ± 1.66	−0.29	−1.20 to 0.59	0.522
diffREC (mm) [mean ± SD]	−0.10 ± 0.99	0.20 ± 0.79	0.33	−0.55 to 1.20	0.465
diffCAL (mm) [mean ± SD]	−3.60 ± 1.51	−3.70 ± 1.83	−0.06	−0.94 to 0.82	0.895
diffTP (mm) [mean ± SD]	0.32 ± 1.01	−0.08 ± 1.08	−0.38	−1.30 to 0.51	0.404

Abbreviations: CAL, clinical attachment loss; d, Cohen's D; diff, difference between the baseline and 1 year; PD, probing depth; *p*‐value, Welch two sample *t*‐test; REC, recession; SD, standard deviation; TP, location of papilla.

## 4 DISCUSSION

The aim of the present study was to compare the efficacy of healing of intrabony periodontal defects filled with two different biomaterials, porcine‐origin collagenated cortico‐cancellous xenogenic particulate bone substitute and cortico‐cancellous heterologous bone gel, in conjunction with papilla preservation procedures. In contrast to standard superiority studies (Tu et al., 2006), our study was designed as a noninferiority study, with the null hypothesis stating that the change in CAL, PD, REC, and TP between baseline and the 12‐month follow‐up would not be worse after defect filling with bone gel as compared to a solid particulate bone substitute. Both biomaterials were found to be effective, with the bone gel tested being noninferior in all primary and secondary clinical outcomes 1 year after surgery. To our knowledge, this is the first noninferiority study to evaluate the clinical outcomes of prehydrated collagenated bone gel versus collagenated xenogenic bone granules applied to periodontal defects. However, since no histomorphometric evaluation was performed, it remains unclear to what extent the application of each material affects the soft‐to‐hard tissue ratio and the regeneration of the periodontal structure.

Compared to deproteinized bovine bone mineral, which is known to be the most used xenogeneic bone material, the investigated porcine bone xenograft was subjected to a lower temperature treatment (up to 130°C), which made it possible to preserve its structure and composition of collagen and hydroxyapatite. The procedure resulted in a decrease in total porosity (33%), interparticle porosity (21%), and mineral content (65%) compared to standard bovine bone, deproteinized with strong alkalis and organic solvents and treated at a higher temperature of 300°C (total porosity 64%, interparticle porosity 51%, and mineral content 95%). This probably led to a faster remodeling process (Miyauchi et al., 2022) and a higher amount of new bone formation (Lim et al., 2023). For this reason, dual phase xenogenic bone matrix substitutes containing a xenogenic mineral bone phase, and an organic collagen phase represent a gradually resorbable biomaterial for bone and periodontal ligament regeneration. They provide an osteoconductive matrix for new bone growth, while their collagen content acts as a chemoattractant, stimulates the recruitment of mesenchymal stem cells, and promotes early angiogenesis. Prehydration of the collagen‐containing material improves its handling properties, resulting in bone gel consistency. Although the use of prehydrated heterologous bone gel in the treatment of critical size bone defects in rabbit femurs failed to support complete regeneration, the product has not yet been evaluated for periodontal regeneration applications (Falacho et al., 2021). It is not yet known whether dual phase collagen‐containing xenografts can promote periodontal regeneration more effectively than the commonly used pure deproteinized bovine bone minerals, β‐tricalcium phosphate, or hydroxyapatite, although porcine bone with a collagen matrix induced a greater amount of new bone growth compared to the deproteinized bovine bone mineral in animal studies (Lim et al., 2023). This regenerative ability could be similar to that of demineralized freeze‐dried bone allografts or freeze‐dried bone allograft materials, which are not approved in Europe due to regulatory concerns. Although there is a lack of comparative studies in periodontology, collagenized bone grafts appear to show increased remodeling and decreased graft stability when used as a filling material in sinus lifts (Miyauchi et al., 2022). However, they do not show inferior performance in maintaining the alveolar ridge after socket sealing (Fukuba et al., 2021).

In our study, participants received nonsurgical periodontal therapy of the entire dentition, including sites targeted for regenerative procedures, 3 months before surgical intervention. Our protocol differed from the originally described EPP or NIPSA protocols (Moreno Rodriguez & Caffesse, 2018; Moreno Rodríguez et al., 2019), in which the sites associated with the defects to be regenerated were treated 2−3 weeks before surgery by instrumenting only the exposed root surface and the first millimeters of the periodontal pocket (marginal periodontal pocket area). The authors of the original EPP and NIPSA descriptions strictly discouraged conventional scaling and root planing to the bottom of the pocket to preserve any residual fibers, avoid inadvertent curettage, and prevent possible tissue shrinkage. Therefore, the deepest portion of the biofilm‐covered root surface was originally intended to remain untouched. Consequently, scaling and root planing to the bottom of the periodontal pockets in our protocol resulted in shallower periodontal defects (7.8 mm) when compared with studies in which only gentle manipulation of the affected area was performed before surgery (9.6 mm) (Moreno Rodríguez et al., 2019). This may be a partial explanation for the poorer clinical results we obtained in terms of reduction of PD and clinical attachment gain after surgery compared with the results reported by the original authors of the EPP and NIPSA procedures.

We assumed that EPP and NIPSA procedures had the same effect and that the therapeutic outcome was independent of surgical technique. In an additional subanalysis, the results of both procedures were compared (results not shown). It was found that the assessment of treatment outcomes based on the same four parameters was not influenced by the specific surgical procedure. Nonetheless, both surgical modalities provided excellent WC throughout the early healing phase and prevented any biomaterial exposure in both the test and control groups. We believe that NIPSA is clearly not suitable for the posterior regions because of several anatomic limitations, such as the exit of the mental nerve, a shallow vestibule, and a short band of keratinized mucosa. Therefore, EPP was used for all surgeries in the premolar and molar regions. However, when the study protocol was proposed, final data on the excellent esthetics of the EPP were not yet available. Therefore, all incisors and canines were treated with NIPSA to avoid scarring. However, later studies have proven that the vertical incision placed away from the bony defect showed impressive healing when sutured, resulting in a high degree of flap integrity during EPP and providing an excellent esthetic outcome (Aslan et al., 2020). Nevertheless, the assumption that the treatment results of the two procedures are equivalent may be incorrect, which is a major limitation of our study.

Another limitation of our study is the lack of a control group without biomaterial. In two recent RCCT studies investigating surgical techniques with and without bone graft substitute, it was found that the addition of bone graft substitute did not improve the clinical attachment gain (Aslan et al., 2020; Moreno Rodríguez & Ortiz Ruiz, 2022). On the other hand, the addition of a bone substitute material improved the stability of the interdental papillae (Moreno Rodríguez & Ortiz Ruiz, 2022), which was also found in our study. Preservation of the interdental papillae is of great importance in achieving a desirable esthetic result in the anterior region. In addition, the presence of the interdental papilla in the posterior region facilitates the feasibility of interdental care techniques by properly closing the interdental space. The absence of significant differences in the position of the papilla tip between the two groups suggests that the preservation of the interdental tissue was well achieved even when a gel‐based bone substitute was used. However, further clinical studies with more specific indications are needed to answer all open questions.

## 5 CONCLUSION

Our results suggest that the use of prehydrated collagen cortico‐cancellous bone gel in conjunction with papilla‐preserving procedures (EPP or NIPSA) results in a comparable reduction in PD and clinical attachment gain and is associated with similar, albeit minor, gingival recession as conventional slowly resorbable solid particulate bone graft substitutes.

## AUTHOR CONTRIBUTIONS

**Tom Kobe**: Methodology, investigation, data curation, formal analysis, writing—original draft. **Katja Povšič**: Data curation, writing—original draft. **Rok Gašperšič**: Conceptualization, methodology, investigation, formal analysis, writing—review and editing, funding acquisition. All authors approved the final version of the manuscript.

## CONFLICT OF INTEREST STATEMENT

The authors declare no conflict of interest.

6

## Data Availability

The author grants access to the data upon reasonable request.
